# DC and AC magnetic fields increase neurite outgrowth of SH-SY5Y neuroblastoma cells with and without retinoic acid

**DOI:** 10.1039/c9ra02001b

**Published:** 2019-06-05

**Authors:** Enad Abed Mahmood Alabed, Martin Engel, Yusuke Yamauchi, Md. Shahriar A. Hossain, Lezanne Ooi

**Affiliations:** School of Biological Sciences, Faculty of Science, Medicine and Health, University of Wollongong Northfields Ave Wollongong NSW 2522 Australia lezanne@uow.edu.au; Illawarra Health and Medical Research Institute Northfields Avenue Wollongong NSW 2522 Australia; Australian Institute for Innovative Materials (AIIM), University of Wollongong North Wollongong NSW 2500 Australia; Department of Biology, College of Science, University of Mosul Ninawa 41002 Iraq; School of Chemical Engineering, The University of Queensland, St Lucia Campus Brisbane QLD 4072 Australia y.yamauchi@uq.edu.au; Australian Institute for Bioengineering and Nanotechnology (AIBN), The University of Queensland, St Lucia Campus Brisbane QLD 4072 Australia md.hossain@uq.edu.au; International Center for Materials Nanoarchitectonics (WPI-MANA), National Institute for Materials Science (NIMS) 1-1 Namiki Tsukuba Ibaraki 305-0044 Japan; School of Mechanical and Mining Engineering, The University of Queensland, St Lucia Campus Brisbane Qld 4072 Australia

## Abstract

It has been suggested that electromagnetic fields could be used to differentiate neurons in culture but how to do this is not clear. We investigated the effect of external magnetic fields (DC and AC MF) on neuronal viability, differentiation, and neurite outgrowth of human SH-SY5Y neuroblastoma cells *in vitro*. A strong low frequency DC MF or a weak AC MF improved retinoic acid-mediated neuronal differentiation and increased neurite length, without any adverse effects on neuronal viability. Even in the absence of the conventional differentiation factor, retinoic acid, DC and AC MF promoted neurite outgrowth. No significant negative effect on cell viability was observed after MF exposure and the DC MF had greater effects on neurite length and branch number than AC MF. Thus, we have identified a novel, simple and cost-effective method that is easy to set up in any cell culture laboratory that can be used to efficiently differentiate neuronal-like cells, using a DC MF without the need for expensive reagents. This research provides a fresh approach to promote neurite outgrowth in a commonly used neuronal-like cell line model and may be applicable to neural stem cells or primary neurons.

## Abstract 2

AC/DC magnetic fields generated from a simple and cost effective electromagnetic system have been used for increased neurite outgrowth in SH-SY5Y cells without negatively affecting the cell viability.

## Graphical abstract

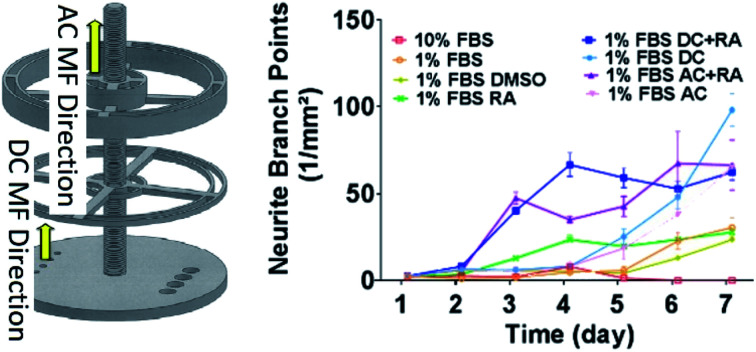

## Introduction

Although it is almost impossible to avoid exposure to electromagnetic fields (EMF) in modern society, the effect of EMF on neurogenesis and neuronal function remains unclear. Thus, understanding the impact of EMF on neurons is required. In addition, within the field of cellular neuroscience, a major challenge is generating cells that faithfully recapitulate neuronal function. Many researchers use neuronal-like cancer cell lines, such as human SH-SY5Y or mouse Neuro2a neuroblastoma cells to provide relatively easy access to a vast number of cells for analysis.^1^ These proliferative cells are commonly differentiated into more neuronal-like cells for functional analyses. To do this, cells can be serum starved and/or treated with an agent, such as retinoic acid (RA) or cyclic adenosine-3′,5′-monophosphate (cAMP) to promote neuronal differentiation.^1^ Although not much is known regarding the influence of EMF on neurogenesis, enhancement of hippocampal neurogenesis in adult mice can occur following exposure to the extremely low-frequency electromagnetic field (ELF-EMF).^2^ The impact of the ELF-EMF on neural stem cells (NSCs) was shown by exposing NSCs to EMF (50 Hz, 1 mT) for 1, 2 or 3 days for 4 h per day, which considerably enhanced NSC proliferation and maintenance.^2^ This ELF-EMF (50 Hz, 1 mT) increased cell division and growth in various cell models^3–5^ and increased proliferation of NSCs.^6,7^ An AC magnetic field (AC MF) was also tested for effects on the neurite outgrowth of PC-12D rat pheochromocytoma cells. Whilst an electric field of 0.2–115 μV m^−1^ had no effect, a 2.2 or 4 mT 50 Hz AC MF stimulated neurite outgrowth.^8^ Despite this, little information is available on the dynamics of EMF exposure on neuronal differentiation. The application of an AC MF varies in orientation and intensity, whereas the DC MF is unidirectional. SH-SY5Y cells are a useful tool as a cell model for neuronal cells but because they are a neuroblastoma cell line, under standard cell culture conditions they continue to proliferate. To promote their differentiation into more neuronal-like cells, serum levels in the culture medium are reduced, in addition to treatment with RA or expensive growth factors. This causes the cells to exit the cell cycle, extend neurites and become terminally differentiated, expressing genes required for neuronal function.^1^ Given the need for a simpler, more cost-effective system, we thus investigated whether a high DC MF could improve the viability and differentiation of SH-SY5Y cells.

## Materials and methods

### Magnetic exposure system

**Fig. 1:**
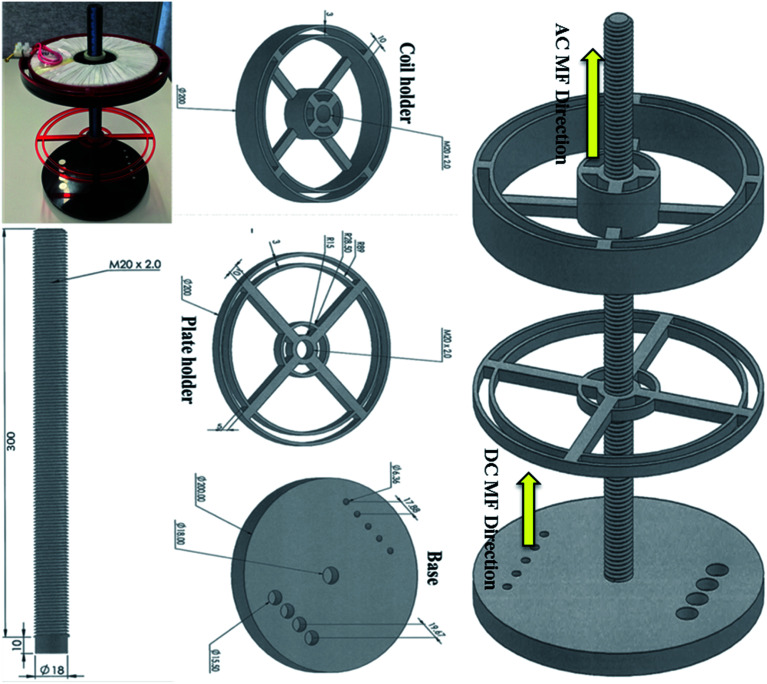
Magnetic exposure system experimental set up. It consists of a base containing two sizes of neodymium cylinder magnets, a base, a rod, a plate holder and a coil holder.

The magnetic field exposure system holder was designed to hold an electromagnet (AC coil) and commercial N52 grade rare earth (N52NdFeB) permanent magnets (for generating DC magnetic field) in close proximity, to obtain a uniform and controlled field gradient by varying the distance between the AC coil and the permanent magnets. The holder was made from Perspex to ensure safety while applying an AC current to the coil. The AC magnetic field exposure system had one circular copper coil (air core) with 400 turns and 10 layers; it was designed at Coast Electrical Industries Pty Limited (Wollongong, Australia) to fit inside the coil holder, which was connected by a 300 mm long rod. The coil was 15 mm long × 160 mm in diameter, and had an internal diameter of 60 mm, as shown in Fig. 1. The wire used in the coil was 1.25 mm in diameter. The coil was connected to a function generator (GFG 200/2100, Good Will Instrument, Taiwan) that applied the frequencies and a power amplifier (model YE5873H; Sinocera Piezotronics, Inc.) to deliver an alternating current of up to 3.6 A. However, to generate 1 mT of electromagnetic field, we applied only 0.36 A by the alternating current. This small current did not produce any coil temperature and hence, thermal changes to the cells. Two sizes (*∅*6.4 × 5 and are *∅*15.6 × 3 mm) of N52NdFeB cylinder magnets were purchased from Chongqing Seatrend Technology and Development. Co., Ltd. (Chongqing, China) and 4 of the larger and 5 of the smaller magnets were placed in parallel on the base of the magnetic system holder. The strength of the MF intensity and frequency for both MFs were assessed using a Gaussmeter (GM-2, AlphaLab, Inc.). This system is just an updated design of our previous work.^9^

### SH-SY5Y cell culture

SH-SY5Y neuroblastoma cells (94030304, Sigma) were cultured in Dulbecco's modified Eagle's medium (DMEM)/F12 pH 7.4 (Life Technologies, 12500096), supplemented with 10% foetal bovine serum (FBS, Bovogen), and 1% penicillin/streptomycin (PS, Life Technologies, 15140-122). The cells were kept in culture for up to 22 passages and maintained at 37 °C in a 5% CO_2_ atmosphere. Cells routinely underwent mycoplasma testing (MycoAlert, Lonza) and were confirmed by Short Tandem Repeat profiling.

### Neuronal differentiation and neurite analysis

The SH-SY5Y cells were plated in 96-well microplates (Greiner, Sigma Aldrich, Sydney, Australia) at 5000 cells per well and then cultured for 7 days. To induce differentiation of the cells, the culture media was replaced with differentiation media (DMEM/F12, 1% PS, 1% FBS, with or without 10 μM all-trans retinoic acid (RA, R2625, Sigma Aldrich)) 24 h after plating, followed by 100% media changes every 48 h. Dimethyl sulfoxide (DMSO) was used as a vehicle control for the RA, and the vehicle control medium was also changed every 48 h. SH-SY5Y cells were treated with different intensities of AC or DC MF (either with or without RA) for 2 days, starting 24 h after plating, and the cells were cultured until day 7 without any further exposure to EMF from day 3. Cell images (four images per well of a 96-well plate) were collected every day using an IncuCyte Zoom live imaging system (Essen Bioscience, Michigan, USA) and analyzed by the IncuCyte software to acquire data on cell confluence (area occupied by cells over the total area), neurite length (total neurite length in mm mm^−2^), and number of neurite branch points (number per mm^2^). Controls were carried out using cells cultured in 10% FBS media. Mean data were calculated from technical replicates of 5 wells for the DC MF and 4 wells for the AC MF. Mean data ± SEM are shown from three independent experimental repeats.

### Cell viability

Cell viability was assessed by the AlamarBlue assay and expressed as a percentage of control cells, as previously described.^10^ In this study, the SH-SY5Y cells were plated into 96 – well microplates at 12 000 cells per well. The cells were cultured in (DMEM)/F12 pH 7.4 supplemented with 10% foetal bovine serum and 1% PS. On the first day the cells were plated a concentration of 12 000/well cells were seeded in the plates. The cells were then treated with AC or DC MF every day for 2 or 3 days, starting 24 hours after plating, depending on the experimental conditions. The cells were cultured for 48 hours without any further treatment with External MF. The fluorescent intensity of the reagent composed from AlamarBlue and the cells was read on the plate reader (BMG-POLARstar Omega) 2 hours after adding the viability reagent to the cells.

### Statistics

The data are shown as the mean ± standard error of the mean (SEM) from at least 3 independent replicates. Statistical differences were identified after confirming normal distribution, by unpaired *t*-test, one-way or two-way repeated measures ANOVA with Tukey's multiple comparisons post hoc, as appropriate.

## Results

### No significant effect or negative impact of DC MF or AC MF on cell viability

**Fig. 2:**
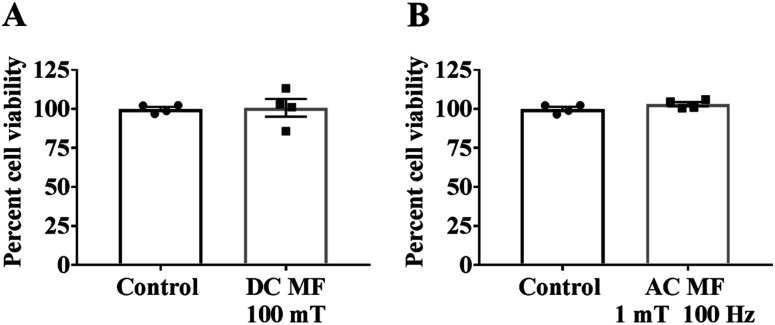
No negative impact of MF on cell viability. Cell viability was assessed by AlamarBlue assay to investigate the impact of (A) a 100 mT DC MF and (B) a 1 mT, 100 Hz AC MF for 4 h per day over 3 days and is shown as a percentage of non-MF-exposed controls. Results show mean ± SEM.

The experimental set up for the magnetic exposure system is shown in Fig. 1. Prior to measuring the effect of DC and AC MFs on neurite outgrowth we first optimised the treatment conditions to ensure there was no significant negative effect on cell viability by any of the treatments. To investigate the impact of DC MF on cell viability, DC MF field strengths were optimized for different treatment times. The 100 mT, 4 h per day condition for 2 days was the best condition for treating cells without any negative impact on cell viability. There was no significant difference between cells treated with DC MF compared to the control (*t* = 0.13, *p* = 0.90, unpaired *t*-test; Fig. 2A). Similarly, AC MF conditions were also optimised and 100 Hz AC MF with a constant magnetic field strength of 1 mT for 4 h per day for 2 days was the best condition for treating cells without any negative impact on cell viability (*t* = 1.55, *p* = 0.17; unpaired *t*-test; Fig. 2B).

### The effects of DC and AC MFs on neurite outgrowth

SH-SY5Y cells were treated with external magnetic fields under either a DC or AC MF to investigate their impact on neurite outgrowth. A range of MF strengths were tested to identify the optimal conditions for treatment. The conditions outlined below represent the best outcomes and involved using an MF strength of 100 mT for the DC MF and 1 mT, 100 Hz for the AC MF. The samples were treated with a 100 mT DC MF or a 1 mT, 100 Hz AC MF for 4 h per day for 2 days. Images of the cells were collected every day until day 7. The effects of external MFs on the SH-SY5Y cells were assessed, including measurement of cell confluency, neurite length and number of neurite branch points.

### Treatment of cells with DC or AC MF for 4 h per day for 2 days increased neurite length and neurite branch points

**Fig. 3:**
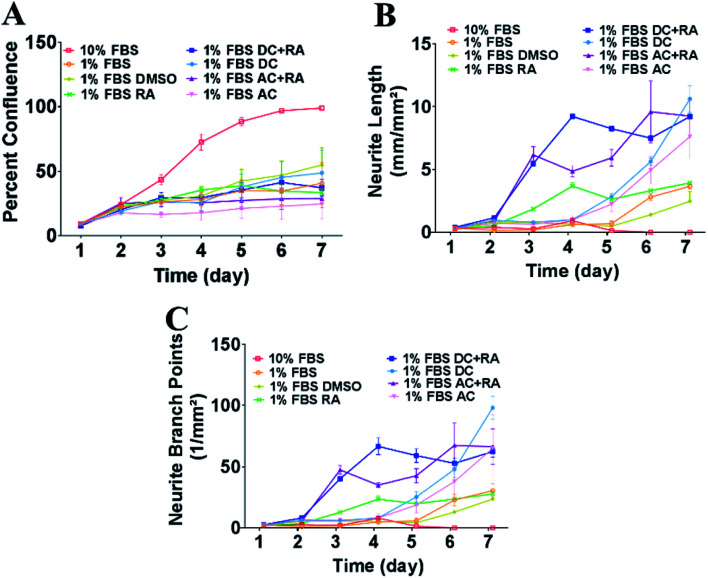
Exposure to DC and AC MFs for 4 h per day over 2 days increased neurite outgrowth and number of branch points in the presence or absence of RA. (A) Percent confluence of the cultures was measured every day from day 1–7. Cells were cultured in 10% FBS (cycling conditions) or 1% FBS to induce neuronal differentiation along with the treatments: vehicle (DMSO), RA, AC MF, DC MF, AC + RA, DC + RA. (B) Neurite length (mm mm^−2^) was measured every day from day 1–7 in the cells cultured in 10% FBS (cycling conditions) or 1% FBS with the treatments: vehicle (DMSO), RA, AC MF, DC MF, AC + RA, DC + RA. (C) Neurite branch points (number per mm^2^) were assessed every day from day 1–7 in the cells cultured in 10% FBS (cycling conditions) or 1% FBS with the treatments: vehicle (DMSO), RA, AC MF, DC MF, AC + RA, DC + RA.

To assess the impact of MFs on neurite outgrowth, the cells were grown in a medium containing 1% FBS supplemented with RA every other day for the 7 day differentiation, and DMSO was used as a vehicle control in these tests. Cells cultured with 10% FBS were used as a control and continued cycling, hence confluence was increased compared with the reduced serum conditions (Fig. 3A) and showed reduced/absent neurite extensions (Fig. 3B and C). Reducing FBS to 1% FBS induced neuronal differentiation and led to a reduction in confluence (***p < 0.001). There were no significant differences in cell confluence for cells cultured under 1% FBS with any of the treatments: vehicle (DMSO), RA, DC MF, AC MF, DC MF + RA, AC MF + RA.

#### (i) DC or AC MF treatment in the presence of RA

There was no significant impact (overall two-way repeated measures ANOVA *F*_(3,8)_ = 0.49, *p* = 0.70) of either AC or DC MFs on cell confluency (Fig. 3A). As expected, RA alone increased neurite length compared to vehicle (DMSO), which became significantly different at day 4 of RA treatment (*p* < 0.01, Fig. 3B). DC MF + RA and AC MF + RA treatment further increased neurite length compared to RA alone, which became significantly different from RA alone from day 3 for both DC MF and AC MF (*p* < 0.001). Neurites increased in length for both types of MF (overall two-way repeated measures ANOVA *F*_(3,8)_ = 37.68, *p* < 0.0001). A post hoc analysis revealed a significant increase in neurite length, starting from day 3 until day 7 for both DC and AC MFs (*p* < 0.001 for days 3 to 7, Fig. 3B). Thus, the impact of treatment on neurite length became significant 24 h after the last treatment. The effect of DC MF + RA on days 3–7 led to a larger increase in neurite length compared to RA alone and compared to AC MF + RA treatment by day 4 (*p* < 0.001). Consistent with the increases in neurite length, the number of neurite branch points were also increased significantly by DC MF + RA or AC MF + RA compared to RA alone, from day 3 until day 7 (overall two-way repeated measures ANOVA, *F*_(3,8)_ = 21.54, *p* = 0.0001); a post hoc analysis identified a significant increase for days 3–7 for both AC MF + RA and DC MF + RA compared to RA alone (*p* < 0.05 for Days 3–7, Fig. 3C). The impact of the AC MF on neurite outgrowth at days 3–7 was lower and more varied than the DC MF treatment (Fig. 3C).

#### (ii) DC or AC MF treatment in the absence of RA

To assess the effect of MF in the absence of RA, RA was omitted from the cell culture medium and the effects of MF on neurite length and number of branch points were assessed as above. In these experiments MF was applied to cells cultured in a medium containing 1% FBS. Both DC and AC MF had similar effects on neurite outgrowth, even without RA. Neither type of MF had an effect on cell confluency (overall two-way repeated measures ANOVA, *F*_(3,8)_ = 11.34, *p* = 0.0030, Fig. 3A). Even in the absence of RA, DC MF and AC MF were able to increase neurite length compared to RA, which became significantly different from RA alone at day 7 (*p* < 0.001). Neurite length increased significantly with either type of MF compared with control (overall two-way repeated measures ANOVA, *F*_(3,8)_ = 16.92, *p* = 0.0008). Post hoc analysis identified a significant increase starting from day 5 until day 7 for the DC MF (*p* < 0.05 for days 5–7, Fig. 3B), and from day 6 to day 7 for the AC MF (*p* < 0.05 for days 6–7, Fig. 3B). Thus, the effect of treatment became significant 3 days after the last treatment day for DC and 4 days for AC. Consequently, the DC MF was marginally better at increasing neurite length than the AC MF, with a significant effect earlier in the differentiation process. However, the effects of AC MF were similar to DC MF by day 7 (Fig. 3B). Even in the absence of RA, DC MF and AC MF were able to increase neurite branch points compared to RA, which became significantly different to RA alone from day 5 for DC (*p* < 0.05) and day 6 for AC (*p* < 0.05). Consistent with the findings on neurite length, the number of neurite branch points increased significantly compared to RA alone at days 5–7 for DC while the impact of AC MF was not significant until days 6–7 (overall two-way repeated measures ANOVA, *F*_(3,8)_ = 6.347, *p* = 0.02); post hoc analysis identified a significant increase starting on day 5 for DC MF (*p* < 0.05, Fig. 3C) and on day 6 for AC MF (*p* < 0.05).

#### (iii) Comparision of DC or AC MF treatment with and without RA

Neurite length in DC MF + RA was significantly increased compared to DC in the absence of RA between days 3–5 (*p* < 0.001) but by day 6 neurite length was not significantly different. Similarly, neurite length in AC MF + RA was significantly increased compared to AC in the absence of RA between days 3–5 (*p* < 0.001) but by day 6 neurite length was not significantly different, *i.e.* in the absence of RA either DC or AC MF was sufficient to promote neurite length to a similar extent as DC MF + RA or AC MF + RA by day 6. Consistent with the results on neurite length, neurite branch points in DC MF + RA were significantly increased compared to DC in the absence of RA between days 3–5 (*p < 0.05, **p < 0.01, ***p < 0.001) but by day 6 branch points were not significantly different. Strikingly, by day 7 DC in the absence of RA had increased branch points that were significantly increased compared to DC + RA (*p* < 0.01). Neurite branch points in AC MF + RA were significantly increased compared to AC in the absence of RA between days 3–5 (*p < 0.05, **p < 0.01, ***p < 0.001) but by day 6 neurite branch points were not significantly different. Thus by day 7 DC MF in the absence of RA significantly enhanced neurite branch points compared to AC MF, DC MF + RA or AC MF + RA. Together our data show that DC MF treatment alone thus provides a simple, convenient method to promote neuritogenesis even in the absence of RA.

#### (iv) Summary of the effect of DC and AC MF on neurite outgrowth

**Fig. 4:**
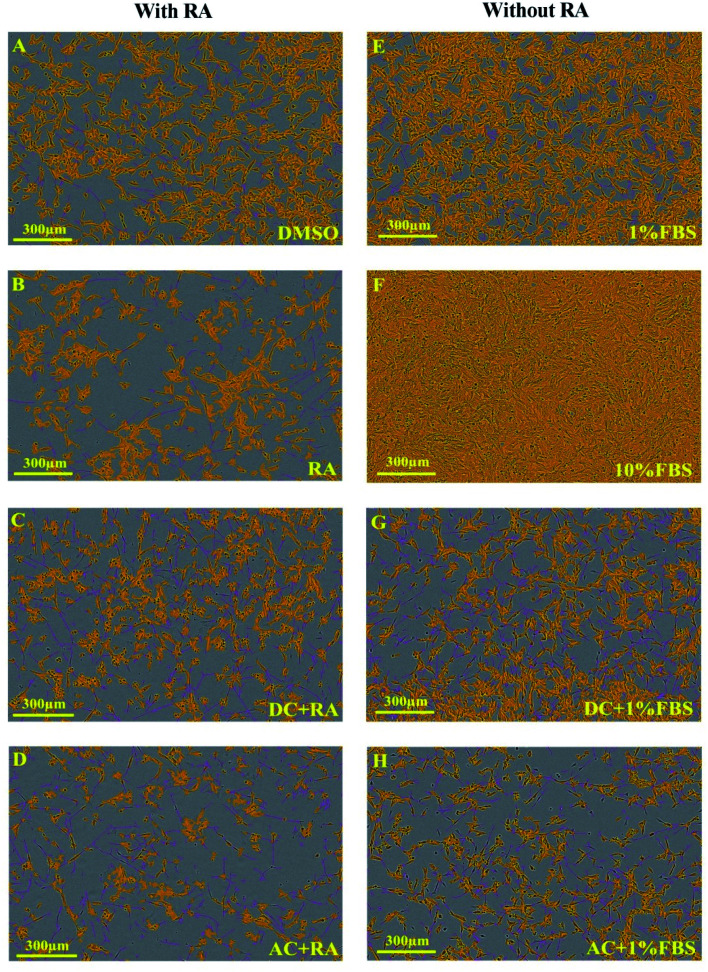
IncuCyte images demonstrating the effects of treating SH-SY5Y cells for 4 hours per day over 2 days with AC and DC MF on neurite outgrowth with and without RA. Images (A–D) are related to the presence of RA. Images (E–H) are associated with the absence of RA. All images were collected on day 7. Yellow refers to the cell body and purple refers to the neurites. Scale bar is 300 μm.

**Fig. 5:**
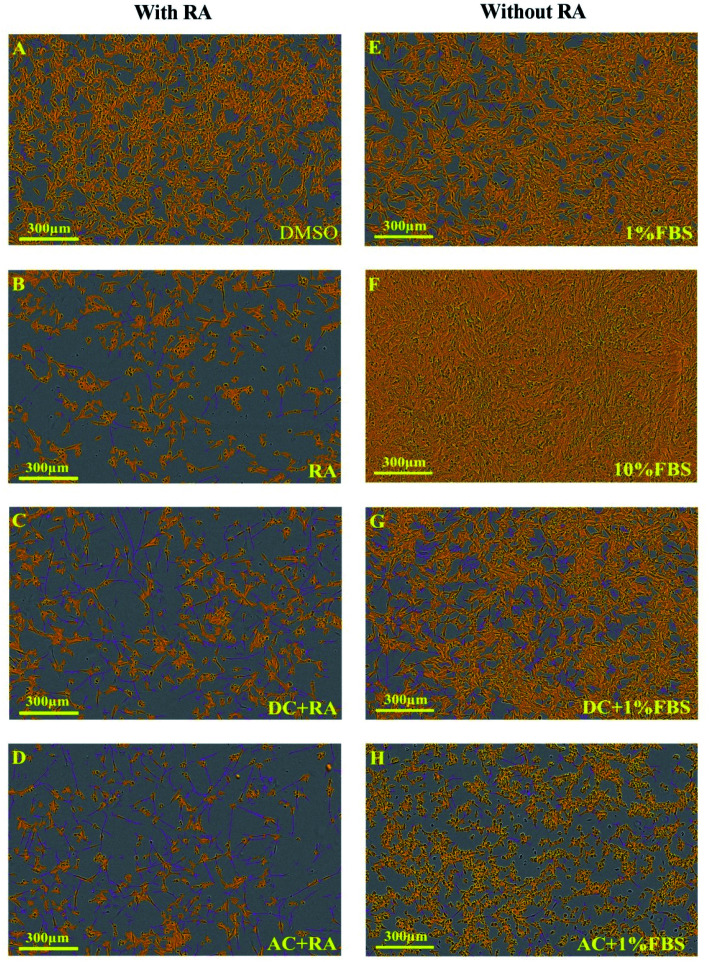
IncuCyte images illustrating the impact of treating SH-SY5Y cells for 4 hours per day over 3 days with DC and AC MF on neurite outgrowth with and without RA. Images (A–D) are related to the presence of RA. Images (E–H) are associated with the absence of RA. All images were collected on day 7. Yellow refers to the cell body and purple refers to the neurites. The scale bar is 300 μm.

Exemplary images of cell confluence and neurite growth are shown in Fig. 4. AC or DC MF for 4 h per day for 3 days treatment was also tested with very similar effects on neurite length, and branch points as for 2 days of treatment. Exemplary images for the effects of these conditions on neurite outgrowth and cell confluence in the presence and absence of RA are shown in Fig. 5. The fold change in neurite length following DC and AC MF treatment in the presence and absence of RA is shown in Table 1. The fold change in number of neurite branch points following DC and AC MF treatment in the presence and absence of RA is shown in Table 2.

**Table 1:** Comparison between the fold change in neurite length of MF compared to control in the 4 hours per day for 2 days and 4 hours per day for 3 days conditions when RA is present and absent (mean ± SEM, *p < 0.05, **p < 0.01, ***p < 0.001, ****p < 0.0001)^a^

Time	4 hours per day for 2 days				4 hours per day for 3 days			
	Presence of RA		Absence of RA		Presence of RA		Absence of RA	
	DC MF	AC MF	DC MF	AC MF	DC MF	AC MF	DC MF	AC MF
Day 1	n.s.	n.s.	n.s.	n.s.	n.s.	n.s.	n.s.	n.s.
Day 2	n.s.	n.s.	n.s.	n.s.	n.s.	n.s.	n.s.	n.s.
Day 3	2.9 ± 0.1***	3.3 ± 0.3****	n.s.	n.s.	4.1 ± 0.4****	3.9 ± 0.7****	n.s.	n.s.
Day 4	2.5 ± 0.1****	n.s.	n.s.	n.s.	2.5 ± 0.1****	1.4 ± 0.1*	n.s.	n.s.
Day 5	3.1 ± 0.1****	2.2 ± 0.2**	4.7 ± 0.9*	n.s.	3.6 ± 0.4****	2.5 ± 0.3****	n.s.	n.s.
Day 6	2.2 ± 0.1****	2.8 ± 0.7****	2 ± 0.1***	1.7 ± 0.2*	2.2 ± 0.2****	3.2 ± 0.4****	n.s.	n.s.
Day 7	2.3 ± 0.01****	2.3 ± 0.3****	2.9 ± 0.3****	2.1 ± 0.5****	2.6 ± 0.2****	2.3 ± 0.2****	3.3 ± 0.2****	2.1 ± 0.5*

a n.s. is non-significant.

**Table 2:** Comparison between the fold change in neurite branch points of MF compared to control in the 4 hours per day for 2 days and 4 hours per day for 3 days conditions when RA is present and absent (mean ± SEM, *p < 0.05, **p < 0.01, ***p < 0.001, ****p < 0.0001)^a^

Time	4 hours per day for 2 days				4 hours per day for 3 days			
	Presence of RA		Absence of RA		Presence of RA		Absence of RA	
	DC MF	AC MF	DC MF	AC MF	DC MF	AC MF	DC MF	AC MF
Day 1	n.s.	n.s.	n.s.	n.s.	n.s.	n.s.	n.s.	n.s.
Day 2	n.s.	n.s.	n.s.	n.s.	n.s.	n.s.	n.s.	n.s.
Day 3	3.1 ± 0.2**	3.7 ± 0.5****	n.s.	n.s.	4.3 ± 0.4****	4.1 ± 0.9****	n.s.	n.s.
Day 4	2.8 ± 0.1****	n.s.	n.s.	n.s.	2.9 ± 0.1****	1.6 ± 0.1*	n.s.	n.s.
Day 5	3 ± 0.3****	2.1 ± 0.3*	n.s.	n.s.	3.8 ± 0.5****	2.6 ± 0.3****	n.s.	n.s.
Day 6	2.2 ± 0.06**	2.8 ± 0.7****	2.1 ± 0.1*	n.s.	2 ± 0.2***	3.1 ± 0.3****	2 ± 0.04*	n.s.
Day 7	2.2 ± 0.1****	2.3 ± 0.5****	3.3 ± 0.3****	2 ± 0.5**	2.5 ± 0.2****	2.2 ± 0.1****	3.8 ± 0.4****	n.s.

a n.s. is non-significant.

## Discussion

One study has reported that only AC MF exposure with and without RA is effective for the cell growth. In this report, ELF and extremely low AC MF (50 Hz–10 μT) has only been applied in the presence and absence of RA for promoting the cell growth in the human neuroblastoma cell line NB69.^11^ Together our data suggest that the effects of DC MF on neuritogenesis were more effective than AC MF. Moreover, whilst 3 days of treatment rather than 2 days had no further benefit with RA present, treating the cells for 2 days rather than 3 days without RA was more effective at promoting both neurite length and number of branch points. Previous studies have reported that cell proliferation in various cell models increased following ELF-EMF exposure.^4,5^ Our cell viability results with AC MF treatment agree with a study that utilised NSCs.^2^ In our study, SH-SY5Y were treated with the same strength of AC MF (1 mT) but at a higher frequency (100 Hz instead of 50 Hz). We also tested 50 Hz in our initial optimisation experiments but found that 100 Hz increased the effects on neurite growth (data not shown).

An impact of MF on reactive oxygen species (ROS) has been identified,^12^ but the influence of MF appears to depend on parameters including exposure time, frequency, intensity of MF, and increasing or decreasing ROS levels within cells.^12^ It is likely that altering MF exposure time affects ROS levels within the cells, which could affect neuronal differentiation and function.

Previous research has also reported that treating NSCs, PC12 pheochromocytoma cells, or dorsal root ganglion sensory neurons with a very low frequency electromagnetic field increased neurite outgrowth,^2,13,14^ but an 1800 MHz radiofrequency electromagnetic field (RF-EMF) reduced the neurite outgrowth of NSCs.^12^ Therefore, this study focused on the impact that low frequency EMF exposure may have on the neurite outgrowth of SH-SY5Y cells. Our findings on SH-SH5Y cells are thus compatible with studies on other neuronal cells^2,13,14^ and suggest that the system we have described could be used to promote neurite outgrowth or neuronal differentiation of different types of neuronal cell lines, neural stem cells or neurons.

A mechanism for inducing neurite outgrowth by exposure to ELF-EMF has been suggested.^2^ In the developing brain, *Ngn1* and *NeuroD,* which are proneuronal basic helix–loop–helix (bHLH) transcription factors, promote neurite outgrowth of NSCs.^15,16^ Expression of these proneural genes, which are crucial for neuronal differentiation and neurite outgrowth, increased after the exposure of NSCs to a 1 mT, 50 Hz AC MF for 4 h per day over 3 days, but exposure to 1800 MHz (RF-EMF) resulted in downregulation of these genes, thereby decreasing neurite outgrowth of NSCs.^17^ Previous studies also reported that the mammalian transient receptor potential channel (TRPC) 1 contributed to neuronal differentiation and proliferation of NSCs and participated in regulating neurite outgrowth.^18,19^ TRPC1 enhanced neurite outgrowth in PC12 cells, whereas TRPC5 decreased it.^20^ Furthermore, exposure of NSCs to an EMF increased intracellular Ca^2+^ by significantly up-regulating the gene expression of TRPC1, and Tuj1+ neurons that differentiated from NSCs, expressed TRPC1, suggested that TRPC1 promotes neurite outgrowth.^2^

A relatively complicated set up is required to apply an AC electromagnetic field to cell cultures. Instruments such as a coil, power amplifier, and function generator are more expensive than permanent magnets, and they can become damaged when connected to an electricity supply. The permanent magnets used as the DC MF source in this study are safer than electromagnetic devices because they are not connected to a power source. Therefore, DC MFs are simpler to set up, safer, and more cost-effective (as they are consumable-free) than AC electromagnetic equipment or consumables such as retinoic acid and growth factors. Permanent magnets can also produce stronger magnetic fields than AC electromagnetic equipment because they can reach 300 mT or higher, depending on the size of the magnet.

Previous studies have investigated the effect of a DC electric field using different cell models.^21–23^ A DC electric field enhanced directional migration and stem cell differentiation, facilitated by calcium influx following DC electric field exposure.^23^ Thus, the mechanism for inducing neurite outgrowth by a DC MF could be the same as for an AC MF and may induce the expression of proneuronal genes, such as *NeuroD* and *Ngn1*, which are crucial for neuronal differentiation and neurite extension. DC MF, as for AC MF,^17^ may also increase the gene expression of TRPC1 to facilitate Ca^2+^ influx. With regards to the reason behind the increased effectiveness of DC over AC and the higher variability in the results for AC MF, we observed that the AC MF increased the temperature of the surrounding area close to the equipment, whereas the DC MF did not, and we speculate that this led to a marginal adverse effect on neurite outgrowth (length and branch points, observed as a reduction in both neurite length and branch points after treatments, these recovered after 5 days).

RA induces neuronal differentiation and limits the proliferation of neuronal-like cell lines and neural progenitors;^24^ however, long periods of differentiation (beyond 8–10 days) with RA are problematic due to the accumulation of proliferating cells.^25^ Since the DC MF promoted neurite extension of neuroblastoma cells in the absence of RA, a DC MF may be able to replace RA in experiments beyond 8–10 days or potentially completely. RA efficiency of neuronal differentiation is also variable in different cell types. Notably, the efficiency of neural differentiation using RA in induced pluripotent stem cells was reported to be much lower than in embryonic stem cells.^26^ Much remains unknown about the effects of MF on neurons and other cells *in vivo*. Transcranial magnetic stimulation (TMS) is becoming widely used clinically (as well as experimentally) to alter neural function and repair neuronal signalling pathways for diverse neurological conditions. Our system could be adapted to uncover the mechanisms by which magnetic stimulation promotes neuritogenesis or neural repair. Harnessing MFs for therapeutic effects *in vivo* is thus an important area of research. Consequently, testing neuronal differentiation in longer experiments, in pluripotent stem cells, and *in vivo* will be important for future research.

## Conclusions

We have identified a simple, cost-effective method of promoting neuronal differentiation of SH-SY5Y cells. Applying external magnetic fields (either DC or AC), with or without the presence of retinoic acid, increased neurite length and the number of neurite branch points without negatively affecting cell viability or cell confluence. In the absence of RA, both types of magnetic fields significantly increased neurite outgrowth following treatment for 4 h per day for two days. Under the parameters used here DC MF was more effective at promoting neurite outgrowth than AC MF.

## Author contributions

M. S. H. initiated this project, and E. A. and M. S. H. designed experiments. E. A. set up the magnetic exposure system and performed cell viability, and neurite outgrowth measurements. E. A., M. E., M. S. H., and L. O. analyzed the data and organized the manuscript. Y. Y., M. S. H. and L. O. supervised the work. All authors discussed the results and contributed to revisions.

## Conflicts of interest

The authors declare no competing interests.

## Supplementary Material
